# Idiopathic Spontaneous Adrenal Hemorrhage in the Third Trimester of Pregnancy

**DOI:** 10.1155/2013/912494

**Published:** 2013-07-31

**Authors:** Narin Nasiroglu Imga, Yasemin Tutuncu, Mazhar Muslum Tuna, Berçem Ayçıçek Doğan, Dilek Berker, Serdar Guler

**Affiliations:** Ankara Numune Education and Research Hospital, Department of Endocrinology, 06100 Ankara, Turkey

## Abstract

Spontaneous adrenal hemorrhage (SAH) is seen in the absence of trauma or adrenal tumor in adrenal glands. The incidence of SAH has been reported from 0.14% to 1.1% and it usually involves the right gland. During pregnancy, idiopathic unilateral SAH has been reported rarely. We present a case which comes to emergency department with an acute abdominal pain and the test results showed spontaneous left SAH.

## 1. Introduction

Spontaneous adrenal hemorrhage (SAH) is an acute hemorrhage of the adrenal gland which occurs in the absence of prior trauma or adrenal tumor. The incidence of SAH has been reported from 0.14% to 1.1% and it usually involves the right gland [1]. During pregnancy, idiopathic unilateral SAH has been reported rarely. The incidence of this condition during pregnancy is unknown [2]. We present a case which comes to emergency department with an acute chest pain and the test results showed spontaneous left SAH. 

## 2. Case Report

A 28-year-old female primigravida in the 34 weeks of gestation with severe acute left upper abdominal pain was admitted to our emergency department. She was hemodynamically stable but had tachypnea. On physical examination, her blood pressure was around 150/90 mm Hg, pulse rate was 96 per minute, and body temperature was 37.2°C. She had pitting edema in the lower limbs. Due to the severe pain in her right flank, she had cold sweats. Hemogram, liver function tests, serum amylase, and lipase were within normal limits. Arterial blood gas parameters were in the normal limits. However, D-dimer level was 2-fold high. Abdominal ultrasound (USG) showed an approximately 10 × 8 cm well-circumscribed and lobulated heterogeneous lesion compatible with hematoma on the left adrenal surgical space. Because of gestation, the patient was asked to the scan abdominal magnetic resonance imaging (MRI). However, she did not accept it. After few minutes, her blood pressure decreased to 90/60 mm Hg and hemoglobin levels to 6.9 g/dL and pulse rate increased to 124 per minute. Blood transfusion and dopamine infusion 5 mcg/min were started and emergency surgery was performed. Prior to unilateral adrenalectomy the caesarean section was performed. In the postoperative period the patient's general condition was normal. Hormonal parameters were in normal ranges. She does not require steroids. The pathology of operation was consistent with adrenal hemorrhage. 

## 3. Discussion

Spontaneous adrenal hemorrhage during pregnancy has rarely been described and may occur in pregnancy in the absence of trauma, tumor, or sepsis [3]. This case highlights an unusual cause of abdominal pain in pregnancy. SAH is an uncommon condition with nonspecific presentation that may lead to acute adrenal crisis, shock, and death if both glands are affected [4]. Pregnancy is associated with adrenal cortex hyperplasia and hypertrophy, which may predispose to venous congestion and hemorrhage. Several risk factors have been associated with it based on case reports. Idiopathic unilateral adrenal hemorrhage is a rare entity that may have an acute presentation, such as massive retroperitoneal bleeding or an adrenal mass [5]. Hemodynamically unstable patients with massive hemorrhage might warrant emergency adrenalectomy and preterm delivery [1]. Adrenal hemorrhage should be considered during pregnancy in condition of abdominal or flank pain. 
